# Resting sympathetic activity is associated with the sympathetically mediated component of energy expenditure following a meal

**DOI:** 10.14814/phy2.13389

**Published:** 2017-09-04

**Authors:** Jacqueline K. Limberg, Katherine R. Malterer, Luke J. Matzek, James A. Levine, Nisha Charkoudian, John M. Miles, Michael J. Joyner, Timothy B. Curry

**Affiliations:** ^1^ Department of Anesthesiology Mayo Clinic Rochester MN 55905; ^2^ Division of Endocrinology Mayo Clinic Rochester MN 55905; ^3^ Thermal and Mountain Medicine Division US Army Research Institute of Environmental Medicine Natick MA 01760; ^4^ Department of Physiology Mayo Clinic Rochester MN 55905

**Keywords:** propranolol, thermic effect of food, *β*‐adrenergic

## Abstract

Individuals with high plasma norepinephrine (NE) levels at rest have a smaller reduction in resting energy expenditure (REE) following *β*‐adrenergic blockade. If this finding extends to the response to a meal, it could have important implications for the role of the sympathetic nervous system in energy balance and weight gain. We hypothesized high muscle sympathetic nerve activity (MSNA) would be associated with a low sympathetically mediated component of energy expenditure following a meal. Fourteen young, healthy adults completed two visits randomized to continuous saline (control) or intravenous propranolol to achieve systemic *β*‐adrenergic blockade. Muscle sympathetic nerve activity and REE were measured (indirect calorimetry) followed by a liquid mixed meal (Ensure). Measures of energy expenditure continued every 30 min for 5 h after the meal and are reported as an area under the curve (AUC). Sympathetic support of energy expenditure was calculated as the difference between the AUC during saline and *β*‐blockade (AUC_P__ropranolol_–AUC_S__aline_, *β*‐REE) and as a percent (%) of control (AUC_P__ropranolol_÷AUC_S__aline_ × 100). *β*‐REE was associated with baseline sympathetic activity, such that individuals with high resting MSNA (bursts/100 heart beats) and plasma NE had the greatest sympathetically mediated component of energy expenditure following a meal (MSNA:* β*‐REE *R *=* *−0.58, *P =* 0.03; %REE *R* = −0.56, *P = *0.04; NE:* β*‐REE *R *= −0.55, *P* = 0.0535; %REE *R* = −0.54, *P* = 0.0552). Contrary to our hypothesis, high resting sympathetic activity is associated with a greater sympathetically mediated component of energy expenditure following a liquid meal. These findings may have implications for weight maintenance in individuals with varying resting sympathetic activity.

## Introduction

The sympathetic nervous system contributes to several components of daily energy expenditure and is important in overall energy balance. Following a meal there is an increase in resting energy expenditure (REE). The difference between fasting REE and postprandial REE is commonly known as the thermic effect of food (TEF). Thermic effect of food is thought to be due to the metabolic demands of digestion, absorption, transport, and storage of nutrients (Acheson et al. 1983) as well as an increase in sympathetic nervous system activity (Patel et al. 1999; Kern et al. 2005; Young et al. 2010; Scott et al. 2013; Taylor et al. 2014).

Consistent with the latter point, pharmacological stimulation of the sympathetic nervous system (e.g., epinephrine, isoproterenol) has been shown previously to initiate a thermogenic response (Stob et al. 1985; Astrup 1986; Staten et al. 1987; Bell et al. 2006). Conversely, systemic administration of adrenergic receptor antagonists (e.g., propranolol) can reduce REE by ~5% (Monroe et al. 2001) and TEF by ~30–40% (Acheson et al. 1983; Tappy et al. 1986); these data suggest that the sympathetic nervous system is responsible for approximately one‐third of the thermogenic response to a meal. Given TEF accounts for up to 10% of total energy expenditure (Reed and Hill 1996), it is reasonable to propose the sympathetic nervous system may play an important role in maintaining energy balance through the sympathetically mediated component of TEF.

Our group has shown that chronic elevations in basal sympathetic nervous system activity (muscle sympathetic nerve activity, MSNA) result in an apparent downregulation of adrenergic receptor responsiveness (Charkoudian et al. 2006). Furthermore, individuals with high resting sympathetic nervous system activity (determined by plasma norepinephrine levels) have a smaller reduction in REE following adrenergic receptor blockade with propranolol (Bell et al. 2001). If the same finding can be translated to the response to a meal, it could have important implications for the role of the sympathetic nervous system in energy balance and weight gain. Taken together, we hypothesized healthy, normotensive individuals with higher resting MSNA would exhibit a smaller reduction in TEF following adrenergic receptor blockade with propranolol. These data would indicate individuals with high resting sympathetic activity have a lower sympathetically mediated component of energy expenditure in response to a meal.

## Materials and Methods

All procedures were approved by the Mayo Clinic Institutional Review Board and all subjects gave written, informed consent. Subjects were asked to complete two study visits in The Clinical Research and Trials Unit (CRTU) of Mayo Clinic's CTSA. Subjects received systemic *β*‐blockade with intravenous propranolol (0.25 mg/kg bolus over 5 min followed by a continuous 0.004 mg/kg/min infusion) (Bell et al. 2001) or a control infusion (equivolume of saline) in random order. We chose propranolol (a *β*‐adrenergic receptor antagonist) because it has been repeatedly shown to reduce energy expenditure via a sympathetically mediated mechanism (Acheson et al. 1983; Tappy et al. 1986; Bell et al. 2001; Monroe et al. 2001). Visits were separated by a minimum of 3 days, and subjects and research personnel were blinded to treatment. Medical personnel (study physicians and/or nurses) were provided with the identity of the study drug on the morning of the study (saline, propranolol) for safety purposes and did not participate in objective data analysis. Subjects were instructed to have no medications on the day of the study (except birth control). Females of childbearing potential were required to have a negative pregnancy test 24‐to‐48 h before participation in each day of the study.

Prior to each study day, a 3‐day standardized balanced diet that supplied 100% of total daily energy expenditure was administered through the CRTU under the supervision of dieticians. The controlled diet consisted of 50% carbohydrates, 20% protein, and 30% fat. Subjects ate all meals at the CRTU or picked up boxed meals that were eaten at home. No other food, snacks, or drinks were permitted. On the third day of the standardized prestudy diet, subjects reported to the CRTU at 1700 h where they consumed an evening study meal and snack (5.5 cal/kg body weight) at the CRTU and remained in the CRTU overnight. At 0700 h on the study day, subjects were moved to the study room and positioned in a supine or semi‐recumbent position. An intravenous catheter for drug infusion was placed in the subject's arm. A 20‐gauge, 5‐cm catheter was placed with real‐time ultrasound guidance in the radial or brachial artery of the contralateral arm under aseptic conditions after local anesthesia (2% lidocaine) and monitored continuously for direct measurements of arterial pressure and sampling of arterial blood. Heart rate was monitored continuously with electrocardiography. Heart rate and blood pressure data are reported as a 15‐min average from baseline and the end of the 5‐h protocol (T300).

Microneurography was performed to obtain MSNA as previously described (Charkoudian et al. 2006). Briefly, the peroneal nerve was localized with transcutaneous stimulation, looking for a motor response. A microneurography electrode was introduced across the skin and placed into the peroneal nerve. Alternatively, the electrode was placed into the peroneal nerve under direct 2‐D live ultrasound guidance using a 12–15 MHz linear probe (Curry and Charkoudian 2011). If study days 1 and 2 took place within 30 days, the contralateral leg was used on the second study day. The recorded signal was amplified 100,000 fold, band‐pass filtered (700 to 2000 Hz), rectified and integrated (resistance‐capacitance integrator circuit, time constant 0.1 sec) by a nerve‐traffic analyzer. Muscle sympathetic nerve activity was expressed as burst frequency (bursts/min) and burst incidence (bursts/100 heart beats). After an appropriate signal was obtained, baseline MSNA was measured for 15 min followed by continuous measurements of MSNA throughout the study. If the nerve signal quality degraded or the signal was lost after baseline measurements were complete, the microelectrode was removed and the study continued without MSNA (baseline MSNA was the primary outcome, *n* = 14, 8M/6F). In most subjects, an acceptable MSNA signal could not be recorded for more than 1 h postmeal because of the sensitivity of the measurement. Therefore, MSNA data is presented for only the first hour postmeal (*n* = 8, 4M/4F).

**Figure 1:**
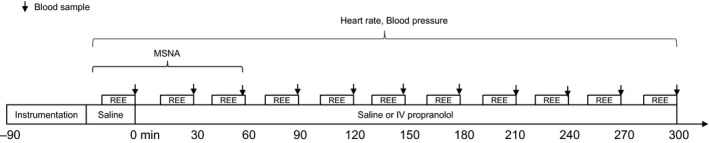
Study day protocol. Subjects completed two study day visits randomized to saline or propranolol. Following instrumentation, baseline measures of heart rate, blood pressure, MSNA, REE, and blood samples were taken (Baseline). Then subjects were given 5‐min to finish an Ensure drink. Energy expenditure was measured for 5 h after the meal for 15‐min periods separated by 15 min of rest and blood samples were drawn every 30 min. MSNA, muscle sympathetic nerve activity; REE, resting energy expenditure.

Resting energy expenditure was assessed at rest by indirect calorimetry with a ventilated hood system (Oxymax H, Columbus Instruments, Columbus, OH). After 15‐min of baseline measurements were obtained, a liquid mixed meal (Ensure^®^, Abbot Laboratories, Columbus, OH) was consumed by the subject within 5 min. The liquid meal supplied 40% of daily energy expenditure (calories) and had a macronutrient composition similar to that of a mixed meal (58% carbohydrates, 14% protein, 28% fat). Energy expenditure was measured for 5 h after the meal for 15‐min periods separated by 15 min. If individuals needed to use the restroom during the testing period they were asked to wait until the start of a 15‐min “break” period between REE measurements and a commode was present in the room in order to resume testing quickly and minimize any potential confounding effect on results. Blood samples for the measurement of glucose, insulin, norepinephrine, and epinephrine were drawn from the brachial artery prior to and every 30 min after the meal and stored on ice until sent for analysis. See Figure 1 for detailed study day protocol.

The thermic effect of food (TEF) was calculated as a change in resting energy expenditure after the meal (measured timepoints: T30, T60, T90, T120, T150, T180, T210, T250, T270, T300) from baseline (e.g., TEF_T30_ = REE_T30_–REE_Baseline_). The area under the curve (AUC) of REE and TEF were also calculated. The difference between AUC during saline infusion and *β*‐blockade (*β*‐REE = REE_Propranolol AUC_–REE_Saline AUC_) was used as a measure of the sympathetic support of REE and TEF (*β*‐REE and *β*‐TEF). In this way, negative values for *β*‐REE are indicative of a fall in REE with propranolol and thus a contribution of the sympathetic nervous system to REE (most negative *β*‐REE = greatest sympathetic support). Changes in REE and TEF following *β*‐blockade were also assessed relative (%) to the control condition (REE_Propranolol AUC_ ÷ REE_Saline AUC_ × 100) to take into account any baseline effect.

Data were compared using a two‐way repeated measures analysis of variance (ANOVA) to test the effect of time, drug (saline, propranolol), and the interaction of time and drug on main (REE and TEF [11 time points], MSNA [5 time points]) and secondary (glucose and insulin [11 time points], epinephrine and norepinephrine [3 time points], heart rate and blood pressure [2 time points]) outcome variables. Paired two‐tailed *t* tests were used to test the effect of drug on REE_AUC_ and TEF_AUC_. The relationships between *β*‐REE or *β*‐TEF and baseline REE, TEF, MSNA, glucose, insulin, epinephrine, norepinephrine, heart rate, and blood pressure were assessed using Pearson Product Moment correlation. Data are reported as Mean ± SEM and *P *≤ 0.05 was considered statistically significant.

## Results

**Figure 2:**
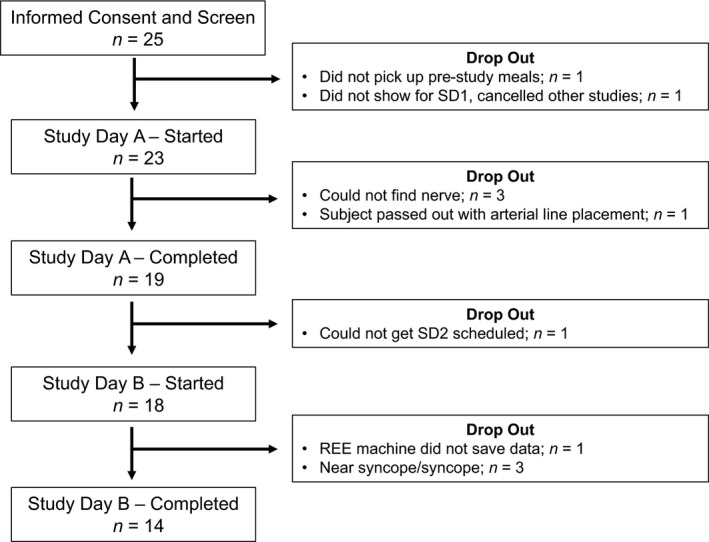
Subject enrollment. Twenty‐five subjects were screened and found eligible for study participation. Twenty‐three subjects began Study Day 1. A clear MSNA signal could not be obtained in *n* = 3 and one subject became syncopal during instrumentation. Of the nineteen individuals with complete data for Study Day 1, one did not return for Study Day 2. Study Day 2 resulted in 3 presyncopal events and 1 equipment error. Complete data are presented from *n* = 14. MSNA, muscle sympathetic nerve activity

Twenty‐five subjects provided informed consent and fourteen subjects (6 female and 8 male; age = 28 ± 2 years, BMI = 24 ± 1 kg/m^2^, MSNA burst frequency = 24 ± 2 bursts/min, MSNA burst incidence = 39 ± 3 bursts/100 heart beats) successfully completed both study visits. See Figure 2.

Heart rate increased following the meal (62 ± 2 to 66 ± 3 beats/min; Main effect of time, *P* < 0.01) and the change in heart rate was attenuated with propranolol (62 ± 3 to 56 ± 2 beat/min; Interaction of time and drug, *P* < 0.01). Mean arterial blood pressure decreased with time (77 ± 3 to 74 ± 3 mmHg; Main effect of time, *P* < 0.01) and the change in blood pressure was greater with propranolol (76 ± 2 to 64 ± 2 mmHg; Interaction of time and drug, *P* < 0.01).

Following consumption of the Ensure drink, circulating concentrations of glucose and insulin were significantly increased (Main effect of time, *P* < 0.01). The immediate (T30, T90) increase in glucose was greater on the saline day (~5–10 mg/dL) when compared to the propranolol day (Interaction of time and drug, *P* = 0.05). Similarly, the immediate increase in insulin (T30, T60, T90) was greater on the saline day (~10–20 pmol/L) when compared with the propranolol day (Interaction of time and drug, *P* < 0.01). See Table 1.

**Table 1:** Plasma glucose and insulin

	Baseline	T30	T60	T90	T120	T150	T180	T210	T250	T270	T300
Glucose (mg/dL)											
Saline	94 ± 2	147 ± 5^b^	146 ± 4^b^	126 ± 4^b^	117 ± 4^b^	114 ± 4^b^	108 ± 4^b^	102 ± 2	102 ± 3	94 ± 2	92 ± 3
*β*‐Blocker	95 ± 2	138 ± 5^a^^,^ b	143 ± 6^b^	121 ± 5^a^^,^ b	120 ± 4^b^	117 ± 4^b^	110 ± 4^b^	102 ± 3	105 ± 4	98 ± 3	100 ± 4^a^
Insulin (pmol/L)											
Saline	4.8 ± 0.6	57.1 ± 10.0^b^	57.5 ± 5.8^b^	39.2 ± 6.1^b^	33.2 ± 4.6^b^	25.3 ± 2.9^b^	20.5 ± 2.7	14.1 ± 2.3	11.3 ± 2.3	7.1 ± 0.9	5.4 ± 1.1
*β*‐Blocker	4.5 ± 0.3	36.3 ± 5.4^a^^,^ b	45.8 ± 6.4^a^^,^ b	28.0 ± 2.7^a^^,^ b	25.1 ± 2.6^b^	22.2 ± 3.4^b^	19.8 ± 3.3^b^	10.9 ± 1.9	12.5 ± 2.9	7.1 ± 1.5	7.7 ± 2.3

a *P *<* *0.05 versus saline.

b *P *<* *0.05 versus T0.

All data are reported Mean ± SEM from *n *= 14, unless otherwise noted (Glucose [Saline, *n *= 13]; Insulin [Saline, *n *= 13–exception T120, T180, T270 *n *= 12]).

Plasma catecholamine concentrations and MSNA data are shown in Tables 2 and 3, respectively. Norepinephrine (*n* = 13) and MSNA (burst frequency, burst incidence; *n* = 8, 4M/4F) increased following consumption of the Ensure drink (Main effect of time, *P* < 0.01). Norepinephrine was lower on the saline day (~40–50 pg/mL) when compared with the propranolol day (Interaction of time and drug, *P* < 0.01). Similarly, MSNA burst incidence was lower on the saline day (~8 bursts/100 heart beats) when compared to the propranolol day (Interaction of time and drug, *P* < 0.01). There were no observable changes in epinephrine (Main effect of time, *P* = 0.07; Interaction of time and drug, *P* = 0.83).

**Table 2:** Plasma catecholamines

	Baseline	T60	T180
Epinephrine (pg/mL*)*			
Saline	31 ± 5	20 ± 3	22 ± 3
*β*‐Blocker	30 ± 5	25 ± 5	31 ± 5
Norepinephrine (pg/mL)			
Saline	143 ± 12	186 ± 20^b^	182 ± 16^b^
*β*‐Blocker	144 ± 13	227 ± 20^a^^,^ b	233 ± 20^a^^,^ b

a *P *<* *0.05 versus saline.

b *P *<* *0.05 versus T0.

All data are reported Mean ± SEM from *n *=* *13.

**Table 3:** Sympathetic activity

	Baseline	T15	T30	T45	T60
Burst Frequency (bursts/min)					
Saline	24 ± 3	25 ± 3	29 ± 3^b^	32 ± 5^b^	33 ± 5^b^
*β*‐Blocker	22 ± 2	24 ± 2	29 ± 3^b^	30 ± 3^b^	35 ± 3^b^
Burst Incidence (bursts/100 heart beats)					
Saline	37 ± 4	37 ± 5	43 ± 5^b^	41 ± 5^b^	46 ± 6^b^
*β*‐Blocker	34 ± 3	45 ± 4^a^^,^ b	50 ± 5^a^^,^ b	55 ± 5^a^^,^ b	55 ± 5^a^^,^ b

a *P *<* *0.05 versus saline.

b *P *<* *0.05 versus baseline.

All data are reported Mean ± SEM from *n *=* *8, unless otherwise noted (Saline T30, *n *=* *7).

**Figure 3:**
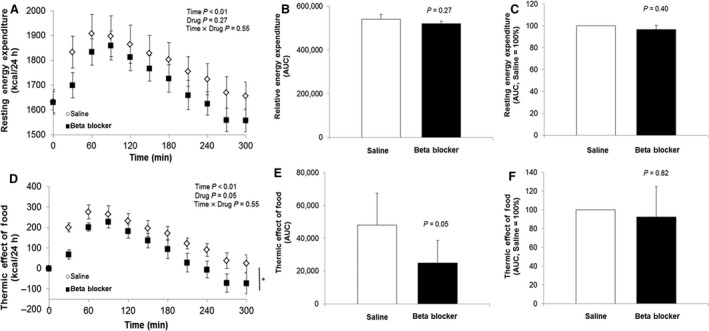
Resting energy expenditure (REE) and thermic effect of food (TEF). REE and TEF during saline infusion (open circles) and during propranolol (closed circles) before (time = −15 min) and for 5‐h after mixed meal (kcal/24 h). REE was increased following the meal (A) and the effect was not altered by propranolol (A–C). TEF was increased following the meal (D) and the response was lower with propranolol when compared to control when assessed as an absolute change (E); however, responses were variable and when data were expressed relative to control, there was no observable effect of propranolol (F). MSNA, muscle sympathetic nerve activity.

Resting energy expenditure increased after the mixed meal (Main effect of time, *P* < 0.01); however, the increase was not different between visits (Interaction of time and drug, *P* = 0.55; AUC *P* = 0.27; %AUC *P* = 0.40, Figure 3A–C). The thermic effect of food (TEF; change in REE from baseline following the meal) also increased over time following the meal (Main effect of time, *P* < 0.01) and TEF was greater on the saline day when compared to the propranolol day (Main effect of drug, *P* = 0.05; AUC *P* = 0.05; Figure 3D–C). However, responses were variable and when data were expressed relative to control, there was no observable effect of propranolol on TEF (%AUC, *P* = 0.82, Figure 3F).

**Figure 4:**
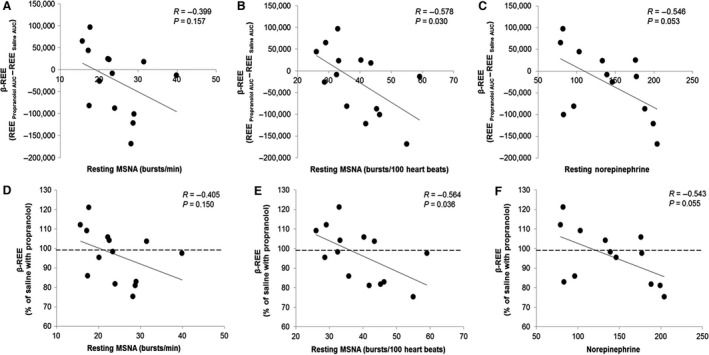
Relationship between baseline sympathetic activity and *β*‐REE. *β*‐REE was related to baseline MSNA and plasma norepinephrine such that individuals with high resting sympathetic activity had the greatest sympathetic contribution of REE following a meal (greatest fall in REE with propranolol = most negative *β*‐REE, (A–C)). Similar conclusions were made when relative (%) changes in REE were assessed (D–F). MSNA, muscle sympathetic nerve activity; REE, resting energy expenditure.

*β*‐resting energy expenditure and *β*‐TEF were related to control REE (*β*‐REE vs REE_Saline AUC_: *R* = −0.77, *P* < 0.01) and TEF (*β*‐TEF vs TEF_Saline AUC_: *R* = −0.70, *P* < 0.01), respectively. *β*‐REE was related to baseline MSNA (Burst Incidence: *R* = −0.58, *P* = 0.03) such that individuals with high resting MSNA had the greatest sympathetic contribution of REE following a meal (greatest fall in REE with propranolol = most negative *β*‐REE, Figure 4A–B). Similar conclusions were made when relative (%) changes in REE were assessed (%REE: Burst Incidence: *R* = −0.56, *P* = 0.04; Figure 4D–E). Any relationships between *β*‐TEF or % TEF and MSNA were not statistically significant (*β*‐TEF: Burst Incidence: *R* = −0.37, *P* = 0.20; %TEF: Burst Incidence: *R* = −0.23, *P* = 0.42). Similar conclusions were made when comparing results with resting plasma norepinephrine (*β*‐REE: *R* = −0.55, *P* = 0.0535; *β*‐TEF: *R* = −0.07, *P* = 0.82; %REE: *R* = −0.54, *P* = 0.0552; %TEF: *R* = −0.04, *P* = 0.89; Figure 4C,F). This may not be surprising, given observed relationships between MSNA and plasma norepinephrine (Burst Incidence: *R* = 0.60, *P* = 0.03). No relationships between *β*‐REE/*β*‐TEF and resting plasma epinephrine were observed (*β*‐REE: *R* = −0.07, *P* = 0.81; *β*‐TEF: *R* = 0.05, *P* = 0.88). No relationships were observed between *β*‐REE/*β*‐TEF and baseline heart rate, blood pressure, or plasma glucose (data not shown). There were significant relationships between *β*‐REE/*β*‐TEF and resting plasma insulin (*β*‐REE: *R* = −0.51, *P* = 0.06; *β*‐TEF: *R* = −0.560, *P =* 0.02) and HOMA‐IR (Homeostatic Model Assessment of Insulin Resistance, calculated as (fasting plasma insulin (mU/L) × fasting plasma glucose (mmol/L))/22.5) (*β*‐REE: *R* = −0.59, *P =* 0.03; *β*‐TEF: *R* = −0.66, *P =* 0.01).

## Discussion

We hypothesized that high resting sympathetic nervous system activity would be associated with a lower sympathetically mediated component of energy expenditure following a meal, based on the idea that chronically high sympathetic nervous system activity would result in downregulation of responsiveness (Charkoudian et al. 2006). Contrary to our hypothesis, we found that young healthy individuals with high baseline MSNA and plasma norepinephrine have a larger sympathetically mediated component of energy expenditure following a meal (*β*‐REE, %REE) when compared with individuals with lower baseline sympathetic activity.

Consistent with previous work (Stob et al. 1985; Schwartz et al. 1987, 1990; Jones et al. 2004), we observed a significant increase in energy expenditure following a mixed meal that was accompanied by an increase in MSNA and plasma norepinephrine. Furthermore, TEF tended to be lower following *β*‐adrenergic receptor blockade, consistent with the work of others (Acheson et al. 1983; Astrup 1986; Thorin et al. 1986; Staten et al. 1987; De Jonge and Garrel 1997; Monroe et al. 2001; Bell et al. 2006). The fall in TEF following propranolol infusion (indicative of *β*‐adrenergic receptor thermogenic responsiveness) has been shown previously to be related to TEF under control/saline conditions (Stob et al. 1985), and our data tend to confirm this. Together with data showing TEF accounts for up to 10% of total energy expenditure (Reed and Hill 1996), we propose the sympathetic nervous system may play an important role in maintaining energy balance (and in body weight homeostasis) through the sympathetically mediated component of TEF.

Our group and others have shown that young lean individuals with high resting MSNA have a lower vascular response to exogenous adrenergic agonists compared to individuals with lower tonic MSNA (Charkoudian et al. 2006). This led us to hypothesize that high levels of basal MSNA would be associated with lower *β*‐adrenergic receptor thermogenic responsiveness to a meal. Consistent with this, obese individuals have an attenuated increase in thermogenesis following adrenergic stimulation (Christin et al. 1989) and decreased thermogenic response to a meal (Stob et al. 1985). Furthermore, obese individuals (Shibao et al. 2007) and individuals after gastric bypass surgery (Curry et al. 2013) exhibit an attenuated fall in REE with sympathetic blockade. In contrast to our original hypothesis, our data show young healthy individuals with high resting MSNA (and plasma norepinephrine) have a *greater* sympathetically mediated component of REE when compared to individuals with low baseline MSNA. These data are more consistent with results from the blood pressure literature, in which higher resting MSNA associated with aging or obesity are linked to a larger role for the sympathetic nervous system in blood pressure control (Jones et al. 2001; Christou et al. 2004; Barnes et al. 2014).

The existing literature provides inconsistent evidence regarding potential relationships between tonic sympathetic activity and energy expenditure. Bell and colleagues have shown individuals with high plasma norepinephrine levels (a crude measure of sympathetic activation) have a smaller reduction in resting energy expenditure following *β*‐adrenergic blockade (Bell et al. 2001). However, other groups found no relationship between plasma norepinephrine and energy expenditure (REE, TEF; Schwartz et al. 1987; Van Gaal et al. 1999) or the fall in REE with propranolol (Monroe et al. 2001), and still others question whether propranolol has any effect on TEF (Morgan et al. 1986; Thorne and Wahren 1989). These discrepancies are likely due to the populations studied, such that the sympathetically mediated component of REE or TEF may only be significant in individuals with increased age, body fat percentage or abdomen‐to‐thigh circumference ratio (older and obese adults; Christin et al. 1989; Schwartz et al. 1990; Bell et al. 2003). Furthermore, variability in responses to *β*‐blockade and the potential “graded” effect of sympathetic nervous system activity may lead to a number of interpretive challenges. In this context, there are both strengths (using the gold‐standard of microneurography and analyzing data as a continuum using linear regression analysis) and weaknesses (studying only young healthy lean individuals) to our experimental approach.

Despite studying only young healthy individuals, we did observe significant relationships between the sympathetic component of energy expenditure following a meal and resting plasma insulin and insulin sensitivity. These data suggest young, healthy individuals with higher fasting insulin and/or suspected decreases in insulin sensitivity have a larger sympathetically mediated component of energy expenditure following a meal when compared to those with lower fasting insulin (<1 *μ*IU/mL) or higher insulin sensitivity (HOMA‐IR <0.2). Of note, the rise in insulin in response to a meal was attenuated with propranolol. It is widely known that *β*‐blockers can attenuate the release of insulin directly via interaction with *β*_2_‐receptors on the pancreas and therefore an attenuated response is not surprising. However, because insulin is sympathoexcitatory (Patel et al. 1999; Kern et al. 2005; Young et al. 2010), the attenuated rise in insulin could have contributed to both a lower increase in MSNA and lower *β*‐REE/*β*‐TEF. Importantly, when we examined the MSNA : Insulin ratio, we found it to be unaffected by propranolol. Therefore, differences in insulin and/or glucose between visits are unlikely to affect data interpretation.

### Experimental considerations

In this study, the increase in heart rate was attenuated and blood pressure fell more in the presence of *β*‐adrenergic blockade and, in fact, there were 3 subjects that did not complete their second study visit due to presyncope. Thus, baroreflex‐mediated increases in sympathetic activity may explain higher MSNA and plasma norepinephrine on the propranolol compared with saline visit. Increased MSNA (sympathetic output to the skeletal muscle vasculature) during feeding presumably reflects a need for peripheral vasoconstriction to maintain blood pressure in response to diversion of blood to the gastrointestinal tract. With this, it is important to acknowledge the sympathetic nervous system is regionally heterogeneous and the largest regional contribution of sympathetic outflow following a meal is directed to the gastrointestinal tract, which cannot be directly assessed by MSNA (sympathetic output to the skeletal muscle vasculature via primarily *α*‐adrenergic receptors) nor arterial norepinephrine (Aneman et al. 1996). However, the assessment of MSNA is perhaps the best practical method available for clinical studies.

The strength of this study was the quantification of sympathetic activity following a meal. The notion that sympathetic “responsiveness” is important is not new; however, when we examined the relationship between the change in sympathetic activity (MSNA burst frequency, burst incidence, plasma norepinephrine) following the meal and *β*‐REE or *β*‐TEF, there were no obvious correlations (*P*‐value range 0.17–0.75). Taken together, our data suggest that baseline sympathetic activity is an important contributor to REE in healthy young humans. However, our results do not support the idea that the sympathetic response to a meal is a major contributor to the TEF component of daily energy expenditure. Furthermore, significant relationships between measures of sympathetic activity and *β*‐REE, rather than *β*‐TEF (change in REE from baseline), suggests the effect of propranolol on REE at baseline (prior to the meal) likely plays a major role in the sympathetic contribution of the response to a meal.

Total energy expenditure can be divided into three primary categories: REE (basal metabolism), TEF, thermic effect of physical activity. Thus, TEF (and in particular, *β*‐TEF) is only one component of overall daily energy expenditure. With this study design, we were unable to test the contribution of MSNA and *β*‐TEF on overall energy balance and/or weight gain. Overfeeding or longitudinal studies would be needed to determine if a greater *β*‐TEF may be protective of long‐term weight gain.

## Conclusion

In conclusion, we have shown that systemic *β*‐adrenergic receptor blockade results in a greater fall in the energy expenditure response to ingestion of a liquid mixed meal in young healthy individuals with higher baseline levels of MSNA. This finding is important to the understanding of the role of the sympathetic nervous system on regulating baseline energy expenditure and energy expenditure in response to a meal. Further studies are needed to determine if high levels of MSNA are protective against weight gain in young healthy individuals.

## Conflict of Interest

There are no competing interests and no relevant conflicts of interest.
